# Prediction of ground vibration due to mine blasting in a surface lead–zinc mine using machine learning ensemble techniques

**DOI:** 10.1038/s41598-023-33796-7

**Published:** 2023-04-21

**Authors:** Shahab Hosseini, Rashed Pourmirzaee, Danial Jahed Armaghani, Mohanad Muayad Sabri Sabri

**Affiliations:** 1 Faculty of Engineering, Tarbiat Modares University, Tehran, Iran; 2 Department of Mining Engineering, Urmia University of Technology, Urmia, Iran; 3 Faculty of Civil Engineering, Centre of Tropical Geoengineering (GEOTROPIK), Institute of Smart Infrastructure and Innovative Engineering (ISIIC), Universiti Teknologi Malaysia, 81310 Johor Bahru, Malaysia; 4 Peter the Great St. Petersburg Polytechnic University, St. Petersburg, Russia 195251

**Keywords:** Environmental sciences, Solid Earth sciences, Engineering

## Abstract

Ground vibration due to blasting is identified as a challenging issue in mining and civil activities. Peak particle velocity (PPV) is one of the blasting undesirable consequences, which is resulted during emission of vibration in blasted bench. This study focuses on the PPV prediction in the surface mines. In this regard, two ensemble systems, i.e., the ensemble of artificial neural networks and the ensemble of extreme gradient boosting (EXGBoosts) were developed for PPV prediction in one of the largest lead–zinc open-pit mines in the Middle East. For ensemble modeling, several ANN and XGBoost base models were separately designed with different architectures. Then, the validation indices such as coefficient determination (R^2^), root mean square error (RMSE), mean absolute error (MAE), the variance accounted for (VAF), and Accuracy were used to evaluate the performance of the base models. The five top base models with high accuracy were selected to construct an ensemble model for each of the methods, i.e., ANNs and XGBoosts. To combine the outputs of the top base models and achieve a single result stacked generalization technique, was employed. Findings showed ensemble models increase the accuracy of PPV predicting in comparison with the best individual models. The EXGBoosts was superior method for predicting of the PPV, which obtained values of R^2^, RMSE, MAE, VAF, and Accuracy corresponding to the EXGBoosts were (0.990, 0.391, 0.257, 99.013(%), 98.216), and (0.968, 0.295, 0.427, 96.674(%), 96.059), for training and testing datasets, respectively. However, the sensitivity analysis indicated that the spacing (r = 0.917) and number of blast-holes (r = 0.839) had the highest and lowest impact on the PPV intensity, respectively.

## Introduction

Mining activities and civil projects are carried out using one of the most important operations, namely rock blasting, as a wide and economical way to rock breakage and displacement of them^1^. In this regard, the rock mass is drilled (drilling operations), and then many blast-holes are charged using explosive materials (blasting operations). Inevitably, blasting operations are caused several side environmental consequences/issues such as flyrock, back-break, dust pollution, air-overpressure, and ground vibration^2–7^. The blast-induced, air over-pressure, ground vibration, and flyrock are the most adverse phenomenon among them^1,8,9^. Therefore, the blasting sites and mine environment must be safe by monitoring and eliminating the adverse effects of the aforementioned consequences. It should be noted that the accurate amount of each phenomenon should be determined/predicted before conducting the operations. The pre-split and power-deck methods can be used to minimization adverse effects^1^.

Ground vibration is the most crucial side environment effect due to bench blasting based on previous investigations^10,11^. The effective parameters on ground vibration should be identified for its prediction/evaluation. The ground vibrations can be measured/recorded based on two different terms: peak particle velocity (PPV) and frequency^12–15^. According to various standards, the PPV is the most famous representative for estimating and evaluating blast-induced ground vibration in surface mines^1,16,17^. The most significant parameters on PPV are the number of blast-holes, hole depth, burden, spacing, powder factor, the charge per delay, and the distance between installed seismograph and blasting bench^18–21^.

In recent decades, many models have been introduced for PPV prediction in mines and open pits. The empirical models have been developed by Davis et al.^22^, Ambraseys and Hendron^23^, Dowding^24^, Roy^25^, and Rai and Singh^26^ for estimation of blast-induced PPV. However, the performance of empirical predictive models is weak and unacceptable. In addition, the empirical equations do not have the ability to accurately predict the PPV values while they must be accurately estimated to overcome the adverse effects. On the other hand, new computational techniques i.e., soft computing (SC) and artificial intelligence (AI) are capable to resolve science and engineering problems in terms of accuracy level^27–30^.

In the field of PPV, a vast range of SC/AI techniques have been proposed for prediction purposes^7,31–35^. For example, Hasanipanah et al.^35^ predicted the PPV values using a genetic algorithm. They concluded that this optimization algorithm can predict PPV values with high accuracy. Imperialist competitive algorithm (ICA) as another optimization algorithm was employed to estimate the value of PPV in the research conducted by Armaghani et al.^6^. They concluded that the ICA algorithm is capable for PPV prediction with high performance. In another study, Taheri et al.^36^ combined artificial neural network (ANN) and artificial bee colony (ABC) to the prediction of PPV; then results were compared to empirical equations. Their results indicated that the performance of the ANN-ABC model is higher than empirical models. Fuzzy system (FS) combined with ICA was introduced in the study conducted by Hasanipanah et al.^13^ to predict PPV. The results of their hybrid model showed that FS-ICA can forecast PPV with a high level of accuracy. Fouladgar et al.^37^ used the cuckoo search (CS) as a novel swarm intelligence algorithm for PPV prediction induced by mine blasting. Additionally, Hasanipanah et al.^38^ established a particle swarm optimization (PSO) technique for forecasting PPV values. In other studies, different techniques such as adaptive neuro-fuzzy inference system (ANFIS) were developed by Iphar et al.^39^ for the estimation of PPV with an acceptable degree of prediction performance. Table 1 summarises the most important studies related to PPV estimation by utilizing the AI and SC techniques.

**Table 1:** Literature review of PPV estimation using AI and SC methods.

References	Year	Model	Inputs	Model performance (R^2^)
Singh et al.^40^	2005	ANN	D, N, HD, B, S, ST, MC, HDI, RDI	0.82
Iphar et al.^39^	2007	ANFIS	DI, CD	0.99
Monjezi et al.^41^	2011	ANN	CD, DI, ST, HD	0.95
Mohamed^42^	2011	ANN, FIS	DI, MC	ANN = 0.94 FIS = 0.90
Khandelwal et al.^43^	2011	ANN	DI, MC	0.92
Fişne et al.^44^	2011	FIS	DI, MC	0.92
Mohamadnejad et al.^11^	2012	SVM, ANN	DI, MC	SVM = 0.89 ANN = 0.85
Ghasemi et al.^45^	2013	FIS	B, S, ST, N, MC, DI	0.95
Masoud et al.^46^	2013	ANN	MC, DI, TC	0.93
Armaghani et al.^47^	2014	PSO-ANN	S, B, ST, PF, MC, D, N, RD, SD	0.94
Hajihassani et al.^20^	2015	ICA-ANN	BS, ST, PF, MC, DI, Vp, E	0.98
Dindarloo^48^	2015	SVM	RD, E, UCS, TS, Js, B, S, HD/B, SC, ST, DPR, DI	0.99
Hajihassani et al.^49^	2015	PSO-ANN	BS, MC, HD, ST, SD, DI, PF, RQD	0.89
Hasanipanah et al.^50^	2015	SVM	DI, MC	0.96
Armaghani et al.^51^	2015	ANFIS	DI, MC	0.97
Ghoraba et al.^52^	2016	ANN, ANFIS	DI, MC	ANFIS = 0.95 ANN = 0.89
Faradonbeh et al.^10^	2016	GEP	B, S, ST, D, HD, PF, MC, DI	0.88
Hasanipanah et al.^53^	2017	CART	DI, MC	0.95
Shahnazar et al.^54^	2017	PSO-ANFIS	DI, MC	0.98
Armaghani et al.^6^	2018	ICA	DI, MC	0.95
Nguyen et al.^55^	2019	HKM-CA	DI, MC, PF, B, S, HD	0.99
Nguyen et al.^56^	2020	SVR-GA	DI, MC, B, S, N	0.99
Zhang et al.^57^	2020	RF,CART,CHAID	B/S, DI, ST, MC, PF, HD	RF = 0.94 CART = 0.97 CHAID = 0.91
Zhou et al.^58^	2020	RF	DI, ST, MC, PF, HD	0.93
Huang et al.^21^	2020	FA-ANN	B/S, DI, ST, MC, PF, HD, RQD, N, SD	0.91
Zhou et al.^12^	2021	GEP-MC	B/S, DI, ST, MC, PF, HD	0.91
Lawal et al.^59^	2021	ANN-MFO	DI, MC, N, HD, RMR	0.97
He et al.^60^	2022	RF-WOA	B/S, DI, ST, MC, PF, HD	0.99
Ragam et al.^61^	2022	XGBoost-RF	N, B, S, HD, D, HD, ST, MC, DI	0.95
Nguyen et al.^62^	2023	SSO-ELM	B, S, PF, MC, f	0.91

*B* Burden, *S* Spacing, *HL* Hole length, *ST* Stemming, *PF* Powder factor, *B* Blastability index, *SVM* Support vector machine, *MC* Maximum charge per delay, *RD* Rock density, *D* Hole diameter, *HD* Hole depth, *BS* Burden to spacing, *N* Number of row, *PSO* Particle swarm optimization, *SD* Sub-drilling, *DI* Distance from the blast face, *TC* Total charge, *RQD* Rock quality designation, *E* Young’s modulus, *ICA* Imperialist competitive algorithm, *Vp* p-wave velocity, *ANFIS* Adaptive neuro-fuzzy inference system, *FIS* Fuzzy inference system, *R*^2^ Coefficient of determination, *UCS* Uniaxial compression strength, *TS* Tensile strength, *Js* Joint spacing, *HD/B* Hole depth-to-burden ratio, *SC* Specific charge, *DPR* Delay per row, *GEP* Gene expression programming, *RMR* Rock mass rating, *f* Rock hardness, *CART* Classification and regression tree, *CHIAD* Chi-square automatic interaction detection, *RF* Random forest, *HKM* K-means clustering, *FA* Firefly algorithm, *WOA* Whale optimization algorithm, *XGBoost* Extreme gradient boosting, *SSO* Sparrow search optimization, *ELM* Extreme learning machine.

An overview of the literature demonstrated that various SC/AI models have been established to estimate the PPV values. Nevertheless, scholars are always looking for models with the highest performance to enhance the accuracy of developed predictive models and decrease the adverse effect of PPV on the environment. Hence, in this study, to increase the accuracy and performance of AI models in the estimation of PPV, an ensemble of XGBoost as well as ANN models are proposed. According to certain research, no machine learning algorithm could ever consistently outperform every other algorithm. In reaction to this assertion, the ensemble learning method was created. Contrary to traditional machine learning approaches, which try to learning a single hypothesis from train dataset, ensemble learning algorithms develop numerous hypotheses and integrate them to solve a specific issue. Ensemble algorithms have resulted in significant improvements and minimized the overfitting issue by integrating numerous learners and fully using these learners. They also offer the flexibility to handle various jobs. Three well-known ensemble approaches include bagging, boosting, and stacking, while there are a few variations and more ensemble algorithms that have been put to use in real-world scenarios^63^. In this way, several publications analysed the performance capability of ensemble models in the various fields such as health science^64^, sport science^65^, agriculture^66,67^, finance^68^, wireless sensor network (WSN)^69^ and geosciences^70^.

The combination of multiple networks and creating an ensemble system can reduce the risk of incorrect results and potentially improve the accuracy and generalization capability. Indeed, an ensemble technique is a robust machine learning method that combines several learners, e.g., ANNs or any other machine learning methods, to improve overall prediction accuracy. In most cases, an ensemble of machine learning methods in comparison to a single learner gives better results^70–72^. This study will introduce a new viewpoint of ensemble modeling to estimate PPV based on two machine learning methods, i.e., XGBoost and ANN models as a stacked generalization technique. For comparison purposes, the performance of the ANNs ensemble method is compared to the XGBoost ensemble method. The more accurate model in forecasting blast-produced PPV will be selected based on the statistical results of all proposed models.

The main research questions are presented as follows:

- How to increase the accuracy of predictive models?
- How is the accuracy level of the model evaluated?
- How is the performance of the proposed model compared to the literature?
- How to measure the validity of the model?
- How is output parameter performance measured against input parameters?

### Case study and data preparation

**Figure 1:**
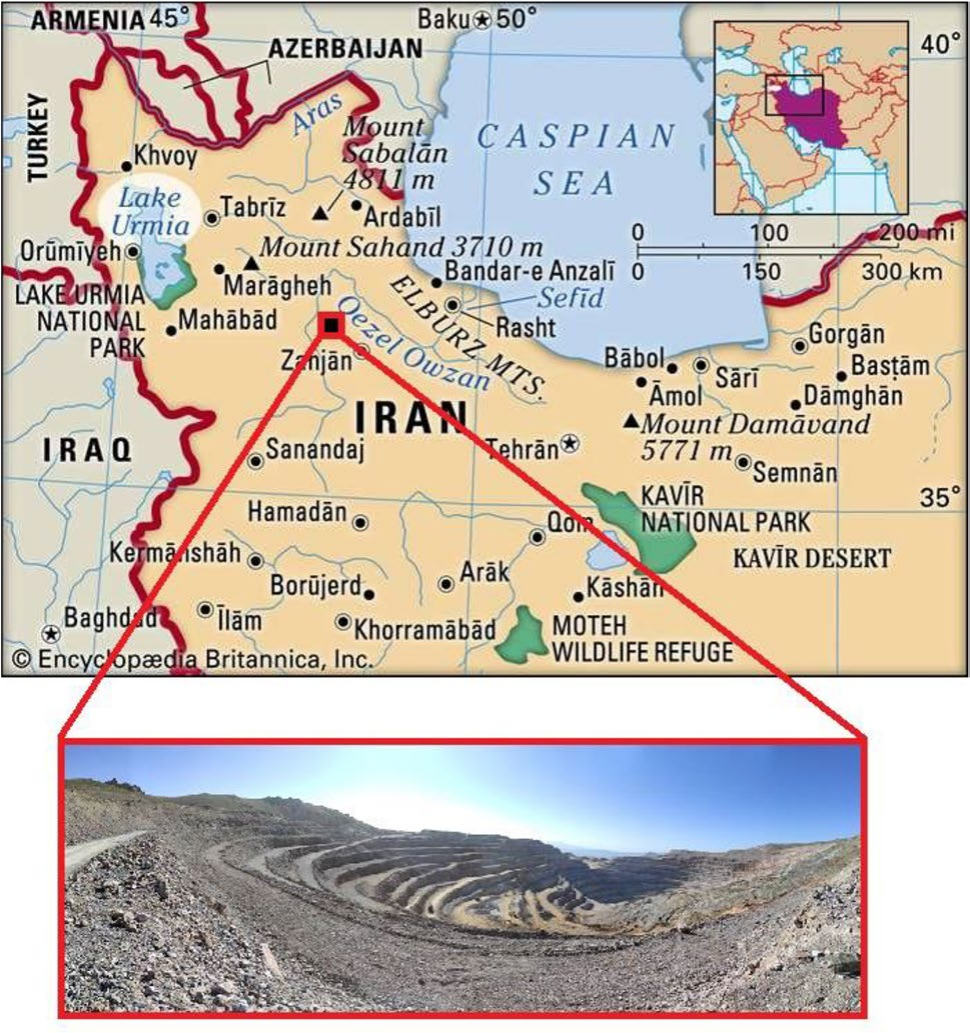
Location of Anguran lead–zinc mine and designed pit^73^ (this figure is modified by EdrawMax, version 12.0.7, www.edrawsoft.com).

This study was focused on the Anguran lead–zinc open-pit mine (Iran), which is located at between longitudes 47° 23′ 27″ N and 47° 25′ 50″ N, and between latitudes 36° 36′ 37″ N and 36° 38′ 04″ N. In addition, the altitude of this mine reported about 2935 m above sea level. Anguran is one of the largest mines in the Middle East (Fig. 1), which is operated with an annual 1.2 Mt extraction rate.

The previous studies considered the blast design parameters as effective parameters on PPV intensity. In this study, we considered the seven blasting pattern design parameters which are used as models’ inputs. These parameters include the number of blast-holes (n), hole depth (l_d_), burden (B), spacing (S), powder factor (q), the charge per delay (Q), and the distance between installed seismograph and blasting bench (d). A total number of 162 blasting rounds were monitored and the effective parameters were measured during operations. The descriptive statistics of the aforementioned parameters are tabulated in Table 2. In the Anguran mine, initiation sequence is inter-row with the time delay of 9 to 23 ms.

**Table 2:** The properties of the parameters and their ranges.

Parameter	Sign	Unit	Minimum	Maximum	Mean	Standard deviation
Inputs						
Number of blast-holes	n	–	10	323	77.28	45.05
Hole depth	l_d_	m	2	12	9.95	2.65
Burden	B	m	3	4.2	4.02	0.33
Spacing	S	m	3.5	6	4.85	0.31
Powder factor	q	Kg/m^3^	0.06	0.75	0.35	0.11
Charge per delay	Q	Kg	43.06	697.72	187.43	88.83
Distance	d	m	305	1167	741.22	248.32
Output						
Peak particle velocity	PPV	mm/s	1.25	28.15	15.08	4.35

The significant relationships between effective parameters and PPV were determined using Pearson cross-correlation. The Pearson test measured the linear correlation of bivariable. The Pearson correlation between parameters and PPV is demonstrated in Table 3, in which the values are calculated in the range of − 1 to + 1. The positive and negative values indicated the positive and negative dependence degree, respectively. Besides, the value of 0 denoted no correlation between the two parameters^74^.

**Table 3:**
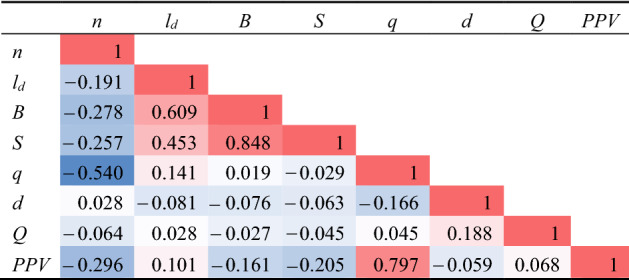
Pearson’s correlation matrix of parameters and PPV.

**Figure 2:**
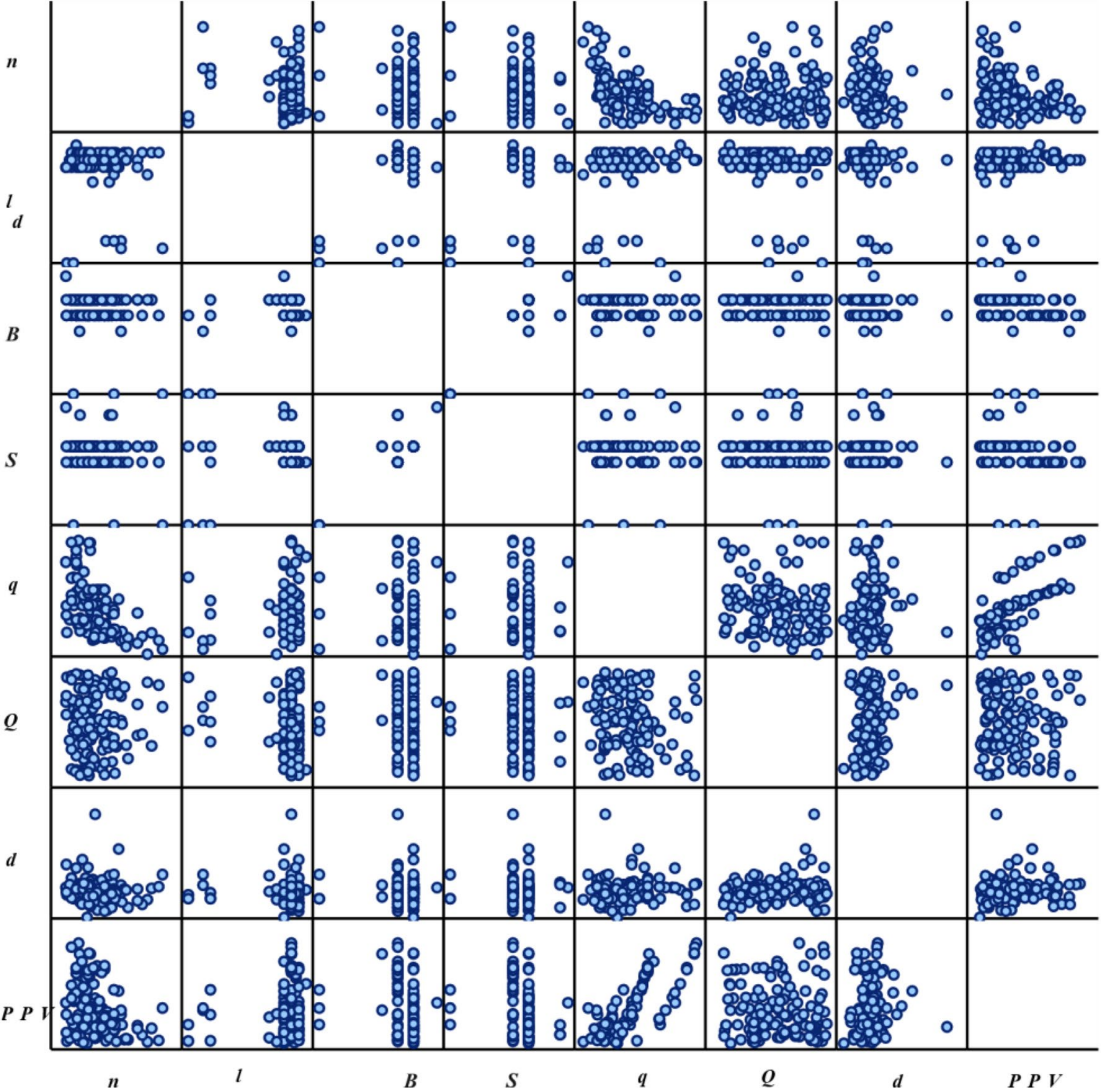
Scatter matrix plot of all parameters considered in this study.

As can be found, the correlation between PPV and PF is high and positive; while PPV and Di have a low and negative correlation. The matrix plot of all parameters is shown in Fig. 2.

## Method background

### Artificial neural network (ANN)

ANN is one of the AI techniques, which first presented in the 1970s. The application of ANN has penetrated various fields of science^75^. A model of ANN is designed based on activities of artificial neural of the human brain. The architecture of an ANN is constructed using the input layer, hidden layer(s), and output layer^76^. Noteworthy, each layer includes many nodes (neurons) which are linked to each other by the weight of the processing components (connections). Input signals, which are the same as input data, are propagated throughout the network using input neurons. Then, input signals pass through the hidden layer(s) and access the output layer. In other words, some calculations are performed during passing signals in each layer and then delivered to the subsequent layer^77–79^. These calculations are formulated in Eq. (1) which simulated the training process of the network^80^

1 $$y = f_{i} \left( {\sum\limits_{i = 1}^{n} {w_{ij} x_{j} + b_{i} } } \right)$$

where *f* denotes activation function, *w* is the weight of connections, *b* indicates bias, and *x* is input data. Notably, the monolayer architecture of the neural network is suitable for simple problems, as well multi-layer architecture is used for complex problems^81^. However, an ANN architecture with two hidden layers for solving engineering problems is usually efficient^75^.

### Extreme gradient boosting (XGBoost)

XGBoost is one of the applicable artificial intelligence techniques, which is firstly introduced by Chen et al.^82^ in 2015. XGBoost, as an AI method, is developed based on the gradient boosting decision. The most important characteristic of this method is creating boosted trees effectively and generating them in parallel. Besides, XGBoost deals with well-known classification and regression problems e.g., Bhattacharya et al.^83^, Duan et al.^75^, Nguyen et al.^84^, Ren et al.^85^, and Zhang and Zhan^86^. In XGBoost, gradient boosting (GB) creates a status under which an objective function (OF) is determined. The optimization of the value of OF is the core of the XGBoost algorithm, which operating to each various optimization technique. Overcoming the problems of data science has made it a robust algorithm. In XGBoost, parallel tree boosting of GB decision tree and GB machine can accurately solve many problems^75,84^. Training loss (L) and regularization (Ω) are the two main components of an OF in this algorithm that defined as follows:

2 $$OF\left( \theta \right) = L\left( \theta \right) \, + \Omega \left( \theta \right)$$

The model performance related to training data is measured using training loss. Notably, the control and overcome overfitting problem as a model complexity is performed by the regularization term. In this regard, the complexity associated with each tree is calculated in several ways; nevertheless, the following formula is widely used to determine the complexity:

3 $$\Omega \left( f \right) = \left( {\gamma \cdot n} \right) + 1/2\lambda \cdot \sum\limits_{j = 1}^{n} {\left( {\omega_{j}^{2} } \right)}$$

where n indicates the number of leaves and $\omega$ denotes the vector of scores on leaves. In XGBoost, the structure score is the OF represented as:

4 $$OF = \sum\limits_{j = 1}^{n} q + \left( {\gamma \cdot n} \right)$$

5 $$q = \left( {G_{j} \cdot \omega_{j} } \right) + \left( {1/2\left[ {H_{j} + \lambda } \right]\omega_{j}^{2} } \right)$$

where q is the best $\omega_{j}$ for a presented structure (a quadratic form). Noteworthy, the $\omega_{j}$ is an independent vector.

### Ensemble modeling

The ensemble of multiple individual learners (base models) is a robust way to enhance the performance and accuracy of artificial intelligence predictive models. In other words, the ensemble model deals with the combination of various models with different results^87^. In general, ensemble modeling includes two components, i.e., an ensemble of base models and a combiner. Training several base models/networks by different subsets of the training data, and employing the different architectures for each of the base models are two common techniques to build the base models^71^, which in current work later method for constructing the base models are used. Also, to the combination of base models, different strategies are proposed where all attempt to reduce the error of estimation.

Generally, combiners are divided into two main groups, i.e., trainable and non-trainable methods. For the combination of the outputs of the base models to achieve a single solution two non-trainable methods, i.e., majority voting and averaging methods, are widely used by scholars, e.g., Barzegar and Asghari Moghaddam^88^, Dogan and Birant^89^, and Krogh and Vedelsby^90^. As such, the mixture of experts and stacked generalization are two trainable combiners that are successfully used in different studies, e.g., Alizadeh et al.^70^, Jacobs et al.^91^, and Wolpert^92^. The trainable combination methods are trained by outputs of base models and expected correct results to predict the final results. The trainable combiners for predicting models that there are complex relations between inputs and targets are more efficient.

**Figure 3:**
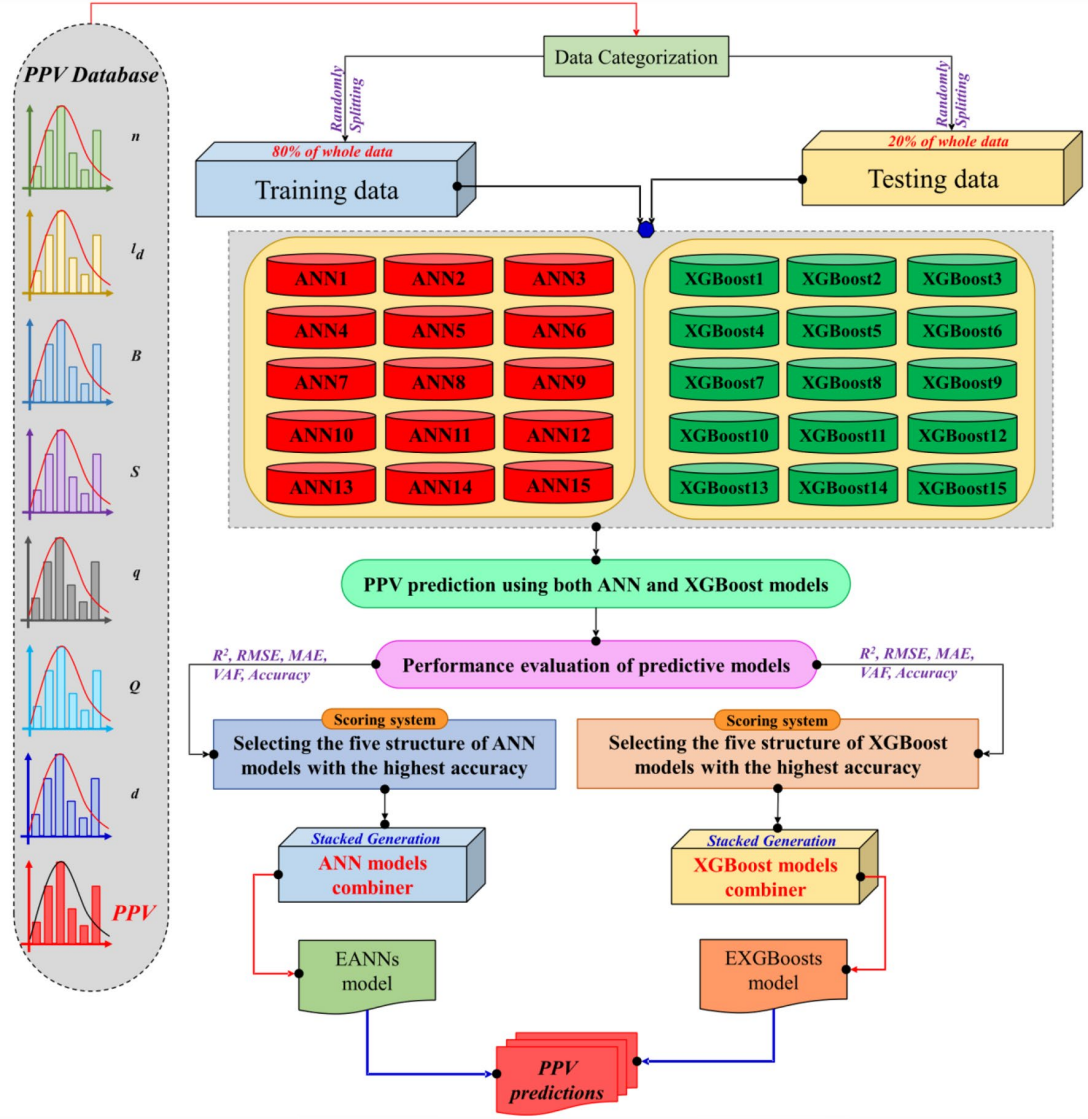
A schematic representation of EANNs and EXGBoosts methods for predicting PPV.

In this study, for each of the methods, i.e., XGBoost and ANN, several models to predict the PPV by stacked generalization technique were combined. In this regard, some ANNs models with a different number of hidden nodes, various activation functions, and different training algorithms for predicting PPV were used. Then top ANNs architectures were combined by the stacked generalization methods to construct the ensemble ANNs that named EANNS model. Notably, various XGBoost models as individual models are developed with different *n*rounds and different maximum depth for PPV estimation, and then top XGBoost models were combined by the stacked generalization technique, which this newly constructed model is called ensemble XGBoosts (EXGBoosts) model. Figure 3 represents the framework of EANNs and EXGBoosts methods, respectively.

### Stacking ensemble model

The stacking model basis is divided into two main phases, which are referred to as level-0 and level-1 structures, respectively. Base models are referred to as level-0, whereas the meta model at level-1 allows base-model predictions to be combined. Estimates provided by base-models are employed throughout the meta-training model's phase. In the case of regression or classification, the predictions result of the basic-models are utilized as inputs and can be of genuine use to the meta-model^69^. The methods of ANN and XGBoost are employed as the base-models in our research. Noteworthy, these models' several separately architectures are each employed individually as meta-learners.

### Pre-analysis of modeling process

This study develops EXGBoosts and EANNs models with seven effective variables and only one output variable to estimate PPV in Anguran lead–zinc mine. In the first step of modeling, all data were normalized in the interval of [0,1], for better network training. Equation (6) was used for normalization of data:

6 $$x_{NORM} = \left( {\frac{{\left[ {x_{i} - x_{min} } \right]}}{{\left[ {x_{max} - x_{min} } \right]}}} \right)$$

where *x*_*norm*_ denotes normalized value, *x*_*max*_ and *x*_*min*_ are the maximum and minimum values, and *x*_*i*_ indicates the input value. In the second step, to present the PPV predictive models, the collected data from the blasting site is randomly divided into two parts, i.e., training and testing datasets. In this regard, 80% of the whole data, namely approximately 130 blasting events, were specified randomly to the training part of models. While the remaining data (approximately 32 blasting events) were used for evaluation of the models' performance.

In the third step, several base models are developed for PPV estimation and the performance of models is compared and evaluated using several statistical indicators such as coefficient determination (R^2^), root mean square error (RMSE), mean absolute error (MAE), the variance accounted for (VAF), and Accuracy (Eqs.7 to 11). These indices are calculated to evaluate the relationship between measured PPV values and estimated one by developed models.

7 $$R^{2} = 1 - \left( {\frac{{\sum\limits_{i = 1}^{n} {(O_{i} - P_{i} )^{2} } }}{{\sum\limits_{i = 1}^{n} {(P_{i} - \overline{P}_{i} )^{2} } }}} \right)$$

8 $$RMSE = \sqrt {\frac{1}{n}\sum\limits_{i = 1}^{n} {(O_{i} - P_{i} )^{2} } }$$

9 $$Accuracy = 100 - \left( \frac{100}{N} \right) \times \frac{{2 \times \sum\limits_{i = 1}^{n} {\left| {O_{i} - P_{i} } \right|} }}{{\left( {O_{i} - P_{i} } \right)}}$$

10 $$MAE = \frac{1}{n}\sum\limits_{i = 1}^{n} {\left| {O_{i} - P_{i} } \right|}$$

11 $$VAF = 100 \cdot \left( {1 - \frac{{var(O_{i} - P_{i} )}}{{var(O_{i} )}}} \right)$$

where *O*_*i*_, *P*_*i*_, and $\overline{P}_{i}$ are measured value, predicted value, and the average of the predicted values, respectively. Also, *n* indicates the number of datasets. However, the value of R^2^, RMSE, MAE, VAF, and Accuracy for the most accurate system are one, zero, zero, 100, and 100, respectively.

### PPV predictive models

#### ANN model

In the present study, for PPV prediction in a surface mine the multi-layer perceptron (MLP) artificial neural network as the most popular structure of ANN was used. The MLP structure contains at least one hidden layer. Hence, the determination of the training algorithm, number of hidden nodes, and hidden layers is a challenge in MLP modeling. In other words, the MLP structure must be designed to train optimally. The feedforward-backpropagation algorithm was used for MLP structure training. In addition, the “trial-and-error” procedure was employed to achieve an MLP model with an optimal structure to predict accurately PPV value. Therefore, 15 different MLP models as base models were developed (Table 4). As can be found, each of the models was trained with different training algorithms, hidden activation functions, output activation functions, and architectures. To determine the optimal architecture, the validation indices of R^2^, RMSE, Accuracy, MAE, and VAF that were formulated in Eqs. (7) to (11) were separately calculated for ANN training and testing datasets. Remarkably, the scoring system proposed by Zorlu et al.^93^ was applied to calculate the rate of each indices for MLP developed models. Table 5 shows the rating indices and ranking of MLP models. Based on results, base model number three with two hidden layers, “tansig” as hidden and output activation functions, and Levenberg–Marquardt (LM) training algorithm is the best base model for PPV prediction. This base model had the 141 total rates out of 150, that the values of (0.948, 0.567, 0.350, 94.767, 94.247) and (0.928, 0.293, 0.487, 92.773, 90.254) are obtained for R^2^, RMSE, MAE, VAF, and Accuracy of training and testing datasets, respectively.

**Table 4:** The base models of ANN and their evaluations.

ANN models	Training algorithm	Number of total hidden nodes	Hidden activation function	Output activation function	Architecture	Training					Testing				
						R^2^	RMSE	MAE	VAF	Accuracy	R^2^	RMSE	MAE	VAF	Accuracy
ANN1	TrainSCG	4	Tansig	Tansig	7-4-1	0.934	0.660	0.428	93.415	91.471	0.724	1.193	0.724	68.725	87.644
ANN2	TrainSCG	7	Logsig	Tansig	7-7-1	0.937	0.693	0.283	93.100	94.829	0.643	1.459	0.756	57.458	85.260
ANN3	TrainLM	10	Tansig	Tansig	7-4-6-1	0.948	0.567	0.350	94.767	94.247	0.928	0.293	0.487	92.773	90.254
ANN4	TrainLM	12	Purelin	Tansig	7-5-7-1	0.883	0.864	0.535	87.290	89.503	0.802	1.395	0.820	77.386	85.371
ANN5	TrainOSS	13	Logsig	Logsig	7-5-8-1	0.932	0.672	0.411	93.213	91.816	0.850	0.492	0.508	84.935	90.061
ANN6	TrainGDX	14	Tansig	Logsig	7-7-7-1	0.939	0.684	0.483	93.774	91.529	0.906	0.754	0.666	89.952	87.247
ANN7	TrainLM	16	Logsig	Logsig	7-7-9-1	0.930	0.643	0.332	92.783	94.510	0.924	0.589	0.360	96.392	90.164
ANN8	TrainGDX	14	Purelin	Purelin	7-9-5-1	0.906	0.799	0.499	90.543	91.588	0.841	0.850	0.622	83.640	87.034
ANN9	TrainSCG	17	Tansig	Purelin	7-9-8-1	0.947	0.677	0.432	94.696	91.873	0.816	0.993	0.606	80.912	88.465
ANN10	TrainGDX	24	Logsig	Logsig	7-11-13-1	0.915	0.913	0.624	88.188	88.413	0.879	1.126	0.985	80.926	79.481
ANN11	TrainSCG	26	Tansig	Tansig	7-11-15-1	0.938	0.619	0.336	93.654	93.553	0.882	1.023	0.586	88.018	90.555
ANN12	TrainGDX	32	Purelin	Tansig	7-15-17-1	0.922	0.680	0.387	92.201	93.114	0.866	0.978	0.392	86.559	88.953
ANN13	TrainLM	37	Tansig	Tansig	7-17-20-1	0.906	0.900	0.628	88.409	87.744	0.763	0.992	0.822	73.105	85.883
ANN14	TrainSCG	39	Purelin	Logsig	7-17-22-1	0.855	0.916	1.501	87.608	64.877	0.765	0.596	1.357	79.748	87.901
ANN15	TrainLM	42	Tansig	Logsig	7-17-25-1	0.913	1.035	0.844	90.418	87.033	0.758	0.981	0.893	75.765	82.328

*LM* Levenberg–Marquardt, *GDX* Adaptive learning rate, *SCG* Scaled conjugate gradient, *OSS* One-step secant.

**Table 5:** Performance of the base models of ANN and their rankings.

ANN models	Training					Testing					Total rate	Rank
	R^2^ rating	RMSE rating	MAE rating	VAF rating	Accuracy rating	R^2^ rating	RMSE rating	MAE rating	VAF rating	Accuracy rating		
ANN1	10	12	9	11	6	2	3	7	2	8	70	9
ANN2	11	7	15	9	15	1	1	6	1	3	69	10
ANN3	15	15	12	15	13	15	15	13	14	14	141	1
ANN4	2	5	5	1	5	6	2	5	5	4	40	12
ANN5	9	11	10	10	9	9	14	12	10	12	106	4
ANN6	13	8	7	13	7	13	11	8	13	7	100	5
ANN7	8	13	14	8	14	14	13	15	15	13	127	2
ANN8	4	6	6	6	8	8	10	9	9	6	72	8
ANN9	14	10	8	14	10	7	6	10	7	10	96	7
ANN10	6	3	4	3	4	11	4	2	8	1	46	11
ANN11	12	14	13	12	12	12	5	11	12	15	118	3
ANN12	7	9	11	7	11	10	9	14	11	11	100	5
ANN13	3	4	3	4	3	4	7	4	3	5	40	12
ANN14	1	2	1	2	1	5	12	1	6	9	40	12
ANN15	5	1	2	5	2	3	8	3	4	2	35	15

#### XGBoost model

Herein, the XGBoost algorithm is used for PPV prediction. Before that, two main stopping criteria, including maximum tree depth and *n*rounds, were determined. These criteria have a significant impact on the performance of models. Similar to MLP networks, the overfitting problem there is also in XGBoost, which is occurred when the tree depth and the *n*rounds are set in the much values. Therefore, the range of [1–3] and [50–200] are considered for the maximum tree depth and *n*rounds. Similar to the ANN, the “trial-and-error” technique was used to determine an XGBoost model with the best performance. As shown in Table 6, the validation indices were computed to evaluate the base models of XGBoost performance. To construct the ensemble of XGBoost, 15 base models with different values of *n*rounds and maximum tree depth were developed. Based on Table 7, 15 base models of XGBoost were evaluated using Zorlu et al.^93^ scoring system. The results were shown that XGBoost base model number two, with the values of 50 and 1 for *n*rounds and maximum tree depth had the best performance in the PPV prediction, which this base model of XGBoost gets the score of 145 out of 150. The validation indices, i.e., R^2^, RMSE, MAE, VAF, and Accuracy were calculated as (0.977, 0.650, 0.402, 97.578 (%), 96.828) and (0.979, 0.536, 0.680, 97.895(%), 96.528) for training and testing datasets, respectively. However, a comparison between top base models of XGBoost and ANN reveals the superiority of the XGBoost method in the prediction of PPV.

**Table 6:** The base models of XGBoost and their evaluations.

XGBoost models	nrounds	Maximum tree depth	Training					Testing				
			R^2^	RMSE	MAE	VAF	Accuracy	R^2^	RMSE	MAE	VAF	Accuracy
XGBoost1	50	1	0.967	0.803	0.526	96.502	95.315	0.962	0.966	0.645	95.760	95.293
XGBoost2	50	2	0.977	0.650	0.402	97.578	96.828	0.979	0.536	0.680	97.895	96.528
XGBoost3	50	3	0.904	1.395	0.876	90.053	92.825	0.899	1.122	0.848	89.762	93.295
XGBoost4	100	1	0.957	0.896	0.629	95.593	94.562	0.952	0.943	0.723	94.777	95.340
XGBoost5	100	2	0.938	1.112	0.764	93.474	93.579	0.937	1.175	0.805	93.248	94.695
XGBoost6	100	3	0.952	0.923	0.626	95.169	94.595	0.968	0.795	0.651	96.645	95.387
XGBoost7	100	1	0.950	0.990	0.66	94.773	94.442	0.943	0.906	0.679	94.238	94.612
XGBoost8	150	2	0.923	1.182	0.771	92.003	93.397	0.882	1.598	1.241	85.342	92.732
XGBoost9	150	3	0.957	0.973	0.631	94.796	94.741	0.959	1.033	0.723	95.330	93.528
XGBoost10	150	1	0.909	1.367	0.861	90.419	92.900	0.900	0.653	0.791	90.020	94.786
XGBoost11	150	2	0.935	1.160	0.762	93.092	93.567	0.951	1.144	0.461	93.577	91.276
XGBoost12	150	3	0.928	1.219	0.828	92.017	93.046	0.952	0.942	0.814	94.961	94.355
XGBoost13	200	1	0.943	1.059	0.708	94.131	93.913	0.924	0.684	0.644	92.392	95.753
XGBoost14	200	2	0.963	0.812	0.568	96.507	94.978	0.904	1.007	0.85	90.304	94.105
XGBoost15	200	3	0.965	0.811	0.547	96.402	95.117	0.909	0.863	0.795	90.701	94.561

**Table 7:** Performance of the base models of XGBoost and their rankings.

XGBoost models	Training					Testing					Total rate	Rank
	R^2^ rating	RMSE rating	MAE rating	VAF rating	Accuracy rating	R^2^ rating	RMSE rating	MAE rating	VAF rating	Accuracy rating		
XGBoost1	14	14	14	13	14	13	7	13	13	11	126	2
XGBoost2	15	15	15	15	15	15	15	10	15	15	145	1
XGBoost3	1	1	1	1	1	2	4	3	2	3	19	15
XGBoost4	10	11	10	11	9	10	8	9	10	12	100	4
XGBoost5	6	6	5	6	6	7	2	5	7	9	59	11
XGBoost6	9	10	11	10	10	14	12	12	14	13	115	3
XGBoost7	8	8	8	8	8	8	10	11	9	8	86	8
XGBoost8	3	4	4	3	4	1	1	1	1	2	24	14
XGBoost9	11	9	9	9	11	12	5	8	12	4	90	6
XGBoost10	2	2	2	2	2	3	14	7	3	10	47	13
XGBoost11	5	5	6	5	5	9	3	15	8	1	62	10
XGBoost12	4	3	3	4	3	11	9	4	11	6	58	12
XGBoost13	7	7	7	7	7	6	13	14	6	14	88	7
XGBoost14	12	12	12	14	12	4	6	2	4	5	83	9
XGBoost15	13	13	13	12	13	5	11	6	5	7	98	5

#### Ensemble model of ANNs (EANNs) to predict PPV

**Figure 4:**
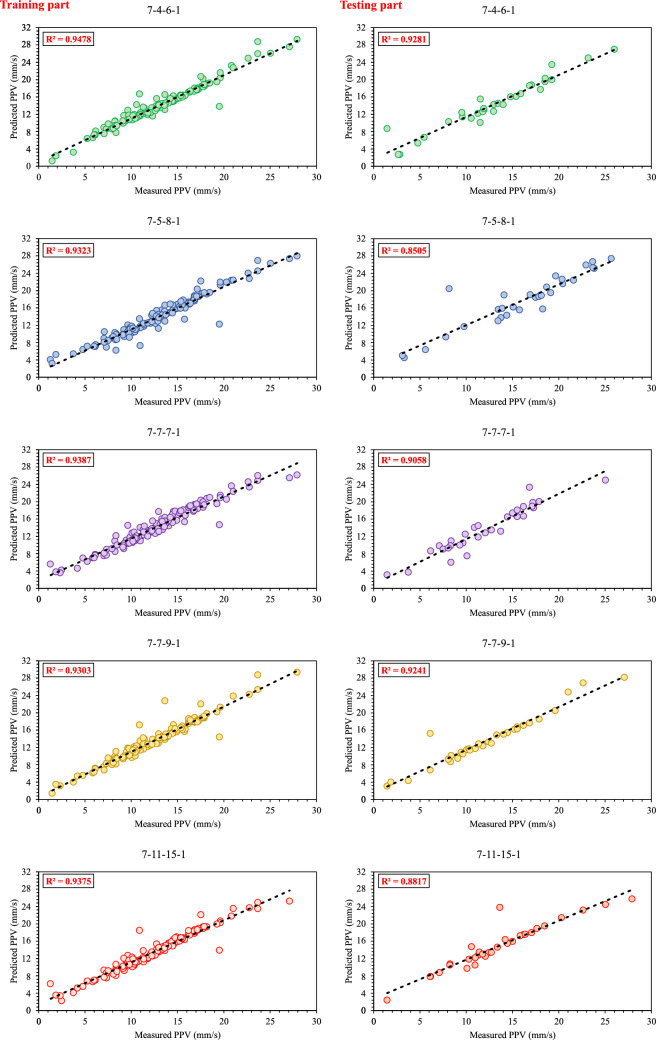
Correlation graph between measured and predicted values of PPV, using five top base models of ANN.

**Figure 5:**
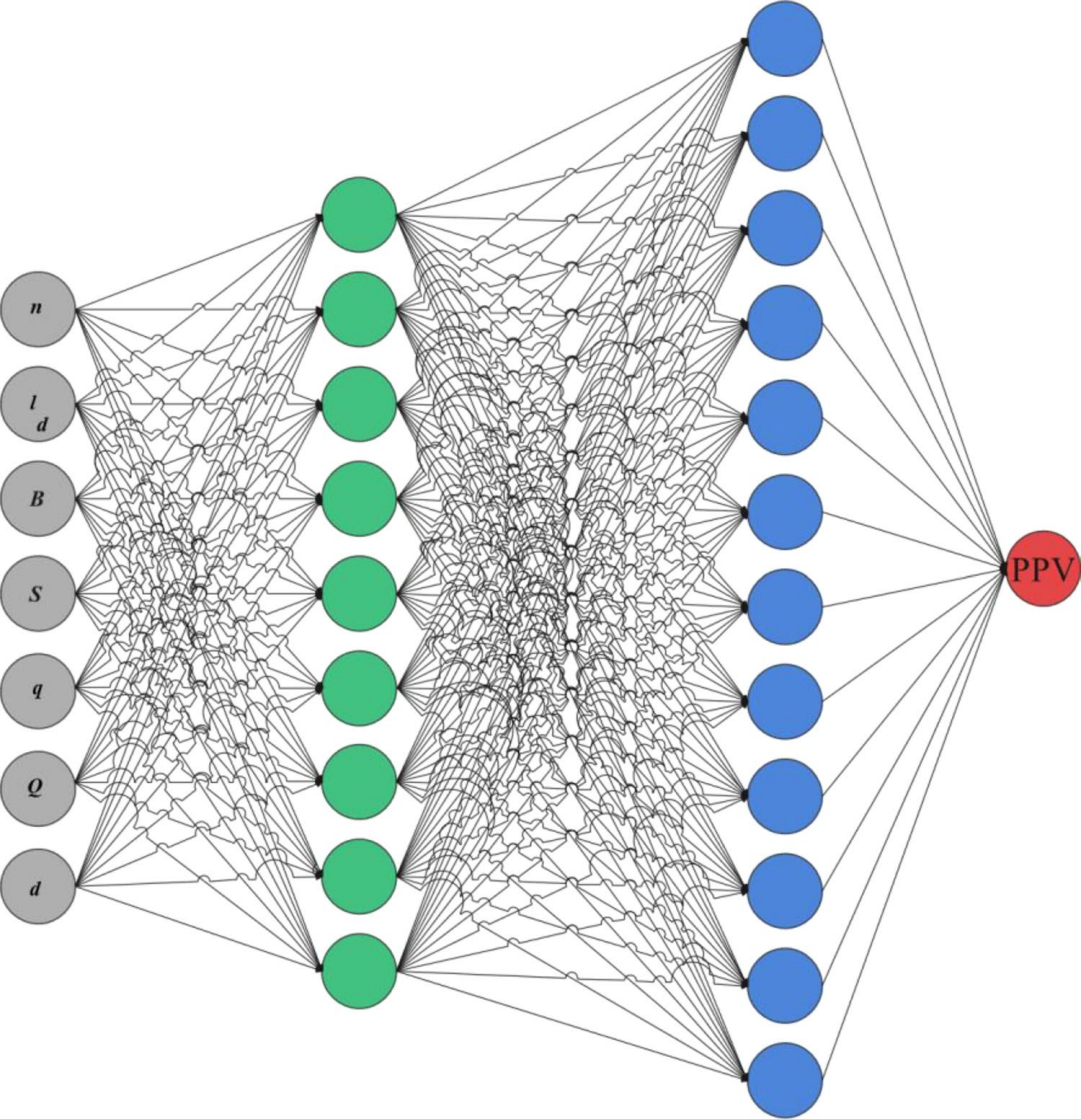
The architecture of the ensemble ANN model for PPV prediction in Anguran mine (this figure is generated by EdrawMax, version 12.0.7, www.edrawsoft.com).

For the ensemble model of ANN, first, 15 base models for ANN are developed, and then after evaluation of the base models, five top base models for combination were chosen, that the scores of these models were 141, 127, 118, 106, and 100 out of 150, respectively. The correlation of measured PPV and predicted ones by five base models are illustrated in Fig. 4. After that, the stacked generalization combination technique was employed to combine the selected base models. For combination, the results of selected base models a feed-forward neural network with sigmoid activation function for hidden layers were used (Fig. 5). The input data of the combiner network consists of seven variables and the target dataset is the measured value of PPV.

**Figure 6:**
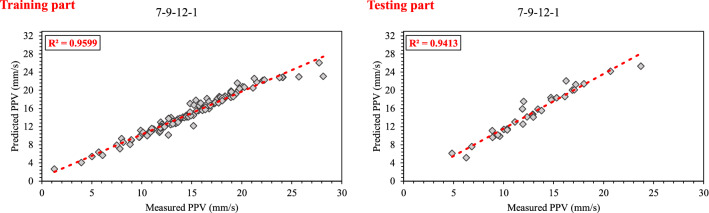
Correlation graph between predicted data (EANNs model) and measured data.

The correlation graph of predicted values using the stacked generalization technique and measured values is illustrated in Fig. 6. The values of (0.960, 0.402, 0.233, 95.963(%), 95.724) and (0.941, 0.189, 0.219, 92.827(%), 95.713) were obtained for both R^2^, RMSE, MAE, VAF, and Accuracy of training and testing datasets, respectively. Results proved that the EANNs model predicts PPV better than individual ANN (base models), so that the EANNs model 41% and 55% improved the RMSE of PPV prediction for training and testing part, respectively, in comparison with the best base model.

#### Ensemble model of XGBoosts (EXGBoosts) to predict PPV

**Figure 7:**
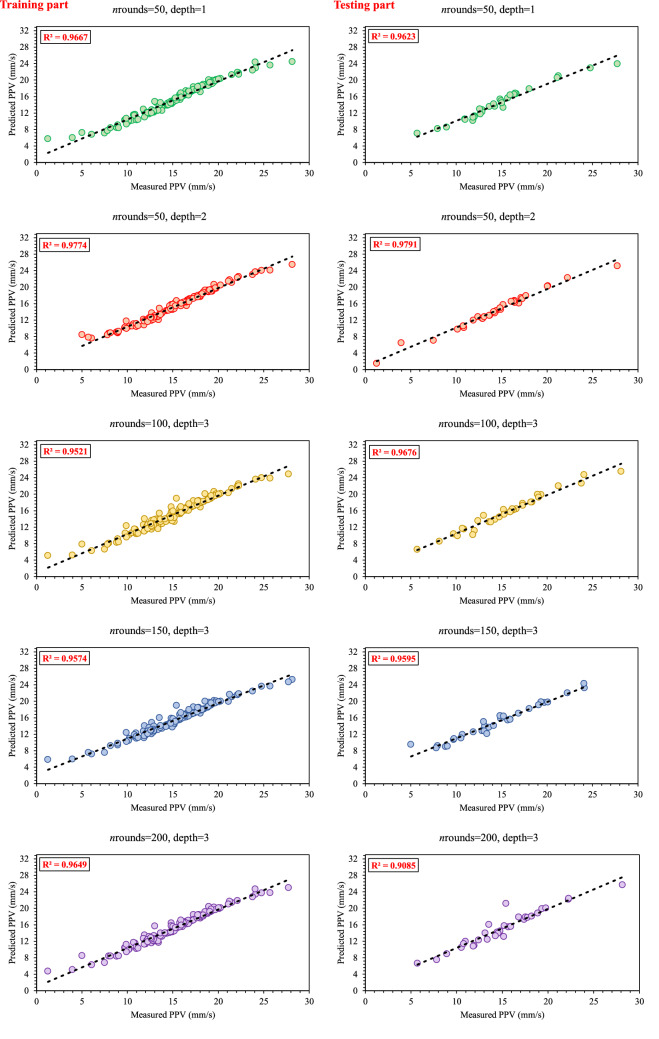
Correlation graph between predicted PPV by various XGBoost base models and measured data.

**Figure 8:**
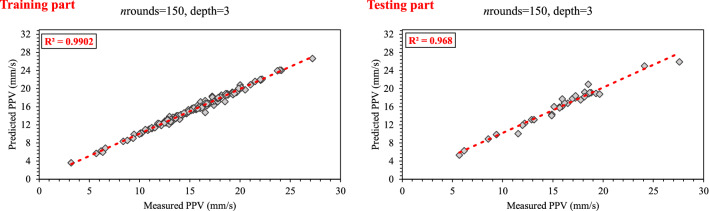
Correlation graph between predicted data (EXGBoosts model) and measured data.

To construct EXGBoosts model for the prediction of PPV, first, several XGBoost models were developed. In this regard, 15 constructed XGBoost models were analyzed, and the five top base models with the highest score were selected. The numbers 145, 126, 115, 100, and 98 were the scores of the five top base models. The EXGBoosts model was structured based on a combination of five XGBoost base models. The base models using stacked generalization technique was combined to predict PPV. Figure 7 showed the correlation of PPV estimations by five XGBoost base models and measured values of PPV. The combiner was structured using a *n*rounds of 15 and a maximum tree depth of three. The results of stacked generalization show, the accuracy of the EXBoosts model in comparison with the best XGBoost base models is better (Fig. 8 and Table 8). To better comparing of the applied methods capability in estimating of PPV value, the performance of developed ANN, EANNs, XGBoost, and EXGBoosts models are tabulated in Table 8. The obtained statistical indices indicated that the EXGBoosts model with the value of (0.990, 0.391, 0.257, 99.013(%), 98.216) and (0.968, 0.295, 0.427, 96.674(%), 96.059) for R^2^, RMSE, MAE, VAF, and Accuracy of training and testing datasets, respectively, represents the highest performance for prediction of PPV among all applied models. Besides, EXGBoosts model 66% and 82% improved the RMSE of PPV prediction for training and testing part, respectively, in comparison with the best base model. The obtained results of performance indices regarding to our model presented in Table 9. This table compares the prediction accuracy and performance level of out proposed approach with three latest research. The results demonstrates that EXGBoost model has more performance capacity in model and estimation of PPV in comparison with the other methods.

**Table 8:** Performance of the ensemble and the best individuals of ANN and EXGBoosts.

Techniques	Part	R^2^	RMSE	MAE	VAF	Accuracy
ANN	Training	0.948	0.567	0.350	94.767	94.247
	Testing	0.928	0.293	0.487	92.773	90.254
EANNs	Training	0.960	0.402	0.233	95.963	95.724
	Testing	0.941	0.189	0.219	92.827	95.713
XGBoost	Training	0.977	0.650	0.402	97.578	96.828
	Testing	0.979	0.536	0.680	97.895	95.528
EXGBoosts	Training	0.990	0.391	0.257	99.013	98.216
	Testing	0.968	0.295	0.427	96.674	96.059

**Table 9:** Accuracy comparison of our proposed technique with other reseach.

Author	Year	Method	R^2^
Huang et al.^21^	2020	FA-ANN	0.91
Zhou et al.^12^	2021	GEP-MC	0.91
Lawal et al.^59^	2021	ANN-MFO	0.97
Ragam et al.^61^	2022	XGBoost-RF	0.95
Nguyen et al.^62^	2023	SSO-ELM	0.91
Proped technique		EXGBoosts	TR = 0.99, TS = 0.97
		EANNs	TR = 0.96, TS = 0.94

*TR* Train, *TS* Test.

It is known that the significance of the estimation of level *l* (where *l* reveals the percentage of estimation) stands the quotient of the number of samples in which the estimations are within l absolute limit of measured values divided by the total number of samples. A common metric for evaluating the best models is P(0.25) ≥ 0.75 or 75%^94^. The level of 25% was used to test model in our study.

In which, where *n* is the number of dataset, *P*_*i*_ denotes the predicted value, and *O*_*i*_ indicates the observed values.

The 25% level estimation of ANN, XGBoost, EANNs, and EXGBoosts are showed in Table 10. As can be seen, the ANN at P(0.25) is not acceptable in validation dataset, but other models is acceptable in both testing and validation datasets. It can be concluded that the ensemble models developed in this study have the highest performance and capability in predicting PPV.

**Table 10:** Estimation level at 25% in testing and validation datasets.

Techniques	Part	P(0.25)
ANN	Testing	76.254
	Validation	33.563
EANNs	Testing	100
	Validation	92.987
XGBoost	Testing	97.255
	Validation	79.654
EXGBoosts	Testing	100
	Validation	100

#### Multiple parametric sensitivity analysis (MPSA)

In this part, a parametric analysis was conducted to specify which influential parameters have the highest impact on the average PPV value. In this regard, a multiple parametric sensitivity analysis (MPSA) was performed that follows the six main steps for applying to the outputs of the system for a specific set of parameters. These steps are as follows:

*Step 1* Selecting the effective parameters to be subjected.

*Step 2* Adjusting the range of input parameters.

*Step 3* Generating a set of independent parameters in the form of random numbers with a uniform distribution for each parameter.

*Step 4* Running the machine learning method utilizing the generated series and calculating the objective function using Eq. (12). The objective function was computed using the sum of square errors between measured and predicted values^95^:

12 $$f_{h} = \sum\limits_{i = 1}^{n} {\left[ {x_{o,h} - x_{c,h} \left( i \right)} \right]^{2} }$$

where *f*_*h*_ denots the objective function value for a particular *PPV*_*t*_ variable *h*; *x*_*o,h*_ indicates the measured values; *x*_*c,h*_(*i*) is the calculated value *x*_*c*_ for variable *h* for each generated inputs; and *n* is the number of variables contained in the random set. In the computation process, the Monte Carlo simulation was used to generate 162 random data for seven effevtive parameters used in this study. At each iteration of the model, the trained models were provided with the newly produced values for one parameter.

*Step 5* Determining the relative importance of effevtive parameters separately using Eq. (13)^95^:

13 $$\delta_{h} = \frac{{f_{h} }}{{x_{o,h} }}$$

In which, *h* is the variable that is used to introduce pairs of effective parameters. The outcomes that were achieved for each of the evaluated parameters were produced by using the technique that was provided to the *PPV*_*t*_ model. Equation (13) had a significant importance in the accomplishment of these results.

*Step 6* Evaluating parametric sensitivity and determining relative relevance of effective parameters using Eq. (14)^95^:

14 $$\gamma = \sum\limits_{h = 1}^{{i_{PPV,max} }} {\delta_{h} }$$

where the *δ*_*t*_ is computed from the first series of dataset (*h* = 1) to the maximum values ($i_{PPV,max}$), which is 162 data for developed model in this study. Table 11 provides a tabular breakdown of the value spectrum that was employed throughout the evaluating of each parameter.

**Table 11:** The range of γ index to determine sensitivity of each parameter^95^.

γ Index	Model parameter sensitivity
γ ≤ 1	Insensitive
1 < γ ≤ 100	Sensitive
γ ≥ 100	Highly sensitive

**Figure 9:**
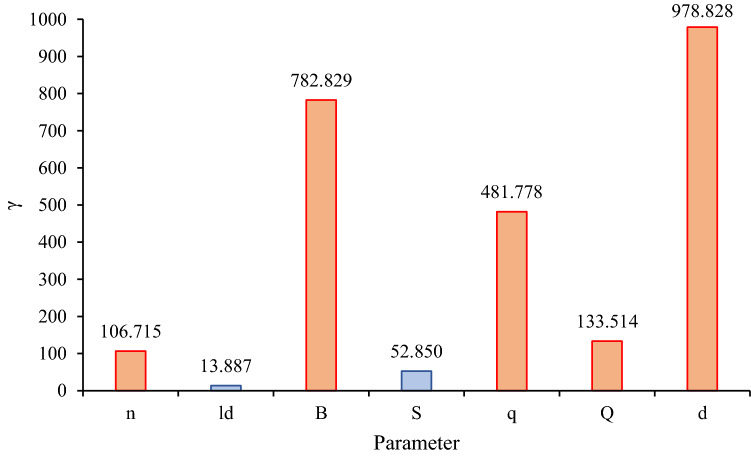
The impact of effective parameters on PPV.

The lower the γ index value for each parameter, the less sensitive the st model is to that parameter, and the higher the γ index, the more sensitive the model is to the parameter under consideration. Table 11 has presented the γ index to evaluate the impact of model parameters and identify the most sensitive parameters. The calculated γ index for each parameter is depicted in Fig. 9. It can be found that the order of the sensitivity of the PPV to the parameters is *l*_*d*_ < *S* < *n* < *Q* < *q* < *B* < *d*. It can be concluded that the PPV is highly sensitive to *d*, *B*, *q*, *Q*, and *n*, as well as sensitive to *S* and *l*_*d*_.

#### Influence of delay sequence on PPV

The seismic energy is what causes the blasting vibrations to be generated, and it also literally symbolizes the problems created to the rock-mass that extends beyond the boundaries of the explosion patch. The blasting pattern design specifications, explosives type and properties, and the physio-mechanical characteristics of the rock-mass all affect how much PPV occurs. The generation of PPV for several experimental implementing blasting has been obtained; the PPV value is reported as 5.12–17.23, 3.91–12.14, and 1.48–5.93 in the delay sequence (row to row) of 9, 15, and 23 ms. It can be concluded that the 23 ms delay between the rows will assist in lowering the PPV, which may be lowered up to a particular value by choosing the right delay sequence in production blast, according to field observations and data analysis.

The superimposition of waveform due to delay sequence refers to the effect of time delays on the coherence of signals. When two or more signals are delayed relative to each other, their waveforms may overlap and interfere with each other, resulting in a composite waveform that may be difficult to interpret. The impact of this effect on the outcome of a result depends on the specific context of the analysis. In some cases, such as in signal processing or communication systems, delay sequences are intentionally introduced to improve signal quality or reduce interference. In these cases, the superimposition of waveforms may be a desirable effect. However, in other cases, such as in physiological or biological signal analysis, the superimposition of waveforms due to delay sequences can lead to a loss of information and inaccuracies in the analysis. For example, in electroencephalogram (EEG) recordings, time delays between signals from different brain regions can result in overlapping waveforms that make it difficult to identify the underlying brain activity.

## Results and discussions

**Figure 10:**
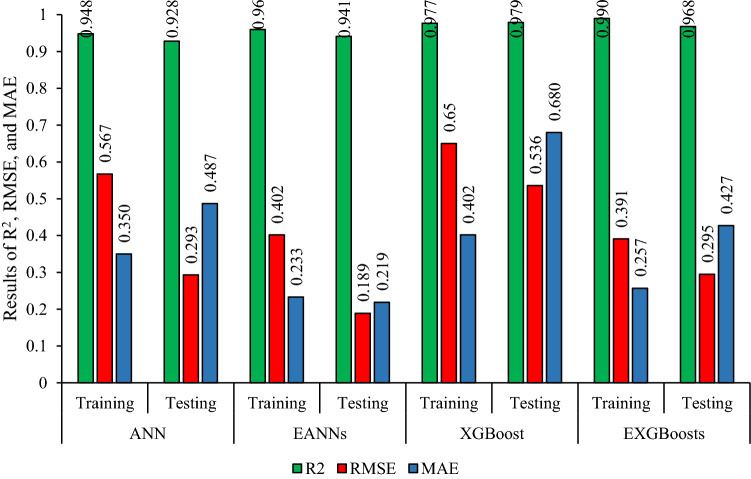
The value of R^2^, RMSE, and MAE for selecting the best model in the predicting PPV values.

**Figure 11:**
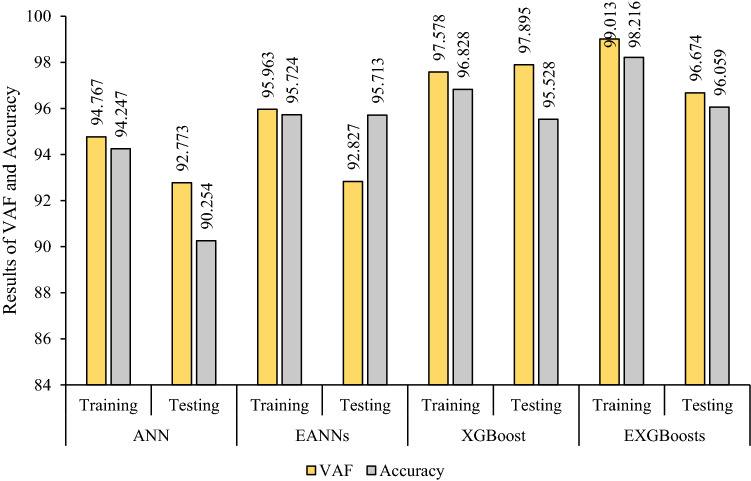
The value of VAF and accuracy for selecting the best model in the predicting PPV values.

This paper was accurately focused on estimation PPV due to mine blasting. In this way, the most effective parameters on PPV variation were identified. Two AI-based models i.e., ANN and XGBoost, were considered for choosing the best between PPV predictive models. For each predictive method, an ensemble model, i.e., EANNs and EXGBoosts, was developed, and the best one was chosen. The obtained results from statistical indicators (R^2^ and RMSE) associated with the best predictive models of ANN, XGBoost, EANNs, and EXGBoosts for training and testing parts were illustrated in Figs. 10 and 11.

The predictive model of EXGBoosts has specified capable of presenting the highest performance prediction level in train and test parts. Therefore, EXGBoosts was found a superior accuracy level regarding statistical indicators values among other predictive models. The R^2^ values of (0.948, 0.977, 0.960, and 0.990) and (0.928, 0.979, 0.941, and 0.968) were calculated for training and testing phases of ANN, XGBoost, EANNs, and EXGBoosts models, respectively. Besides, RMSE values of (0.567, 0.650, 0.402, and 0.391) and (0.293, 0.536, 0.189, and 0.295) were obtained for training and testing parts of ANN, XGBoost, EANNs, and EXGBoosts models, respectively. The EXGBoosts model revealed a maximum performance and minimum system error between other predictive models. In situations where the testing datasets reflect adequate generalizability of predictive techniques, the excellent efficiency of the train phases suggests the success of the learning procedures of the predictive models.

### Benefits and drawbacks of the study

The main benefit of this study is in improving the performance and accuracy of the proposed ANN and XGBoost models. These models separately provide lower accuracy than the ensemble models. Therefore, using the combination of these methods and constructing an ensemble model, it is possible to predict the PPV with acceptable accuracy. Noteworthy, neural network base models each have different results and have uncertainty due to being a black-box. However, the ensemble model solves this problem to an acceptable. This study also has drawbacks. In this study, only two AI models have been used i.e., ANN and XGBoost. However, the number of AI models can be increased to reach maximum accuracy. It should be noted that the number of base models in this study is acceptable; nevertheless, more models can be obtained and run the ensemble model based on them.

## Conclusions

In this study, the PPV induced from bench blasting is studied in Anguran lead–zinc mine, Iran. Considering the crucial importance of the adverse effects of ground vibration in blasting operations, the prediction of PPV at a high level of accuracy is essential. Therefore, this study investigates the ensemble of various artificial intelligence models to construct an accurate model for PPV estimation using 162 blasting datasets and seven effective parameters. For this aim, several ANN and XGBoost base models were developed and the five top base models among them were combined to generate EANNs and EXGBoosts models. To combination of top base models’ outputs and achieve a single result stacked generalization technique was used. The statistical indexes of R^2^, RMSE, MAE, VAF, and Accuracy were used to evaluate the performance of developed models and a scoring system was applied to select the best ANN and XGBoost base models with optimal structure. The results revealed that the EANNs with R^2^ of (0.960, and 0.941), RMSE of (0.402, and 0.189), MAE of (0.233, and 0.219), VAF of (95.963(%), and 92.827(%)), and Accuracy of (95.724, and 95.713) for training and testing datasets, respectively, and EXGBoosts model with R^2^ of (0.990, and 0.968), RMSE of (0.391, and 0.295), MAE of (0.257, and 0.427), VAF of (99.013(%), and 96.674(%)), and Accuracy of (98.216, and 96.059) for training and testing datasets, respectively, were two efficient machine learning ensemble methods for forecasting PPV. Comparison of the results of developed ensemble methods, i.e., EANNs and EXGBoosts, with the best individual models showed the superiority of ensemble modeling in predicting PPV in surface mines. Moreover, EXGBoosts model was most accurate compared to the EANN model. In the final step of this study, the effectiveness of each input variable on PPV intensity is determined using the CA method, which results denoted the spacing has the most impact on PPV. From practical applications, the proposed model can be updated for other engineering fields, specially mining and civil activities. Meanwhile, the ensemble machine learning approach can be applied to improve performance capacity of machine learning techniques and increase the accuracy level of prediction targets. The proposed models can be used to analyze safety data and identify potential hazards, blasting safety zone, and risks in blasting operations. The PPV values can be predicted before blasting operations to check any potential issues or damage to the workers, equipment and surrounding residential area. If the predicted results are higher than those suggested in literature or standards, the blasting pattern/design can be reviewed again to have a predicted PPV values within the suggested safe ranges. Generally, machine learning algorithms can be used to analyze environmental data and monitor the impact of mining operations on the environment.

## Data Availability

The datasets used and/or analysed during the current study available from the corresponding author on reasonable request.
